# *mmCIF Validator*: a comprehensive validation tool for structural biology data files

**DOI:** 10.1107/S1600576726005431

**Published:** 2026-07-24

**Authors:** Deborah Harrus, Genevieve L. Evans, Sreenath Sasidharan Nair, Weslley Morellato Bueno, Jennifer Fleming, Sameer Velankar

**Affiliations:** a Protein Data Bank in Europe, European Molecular Biology Laboratory European Bioinformatics Institute (EMBL-EBI) Wellcome Genome Campus Hinxton, CambridgeCB10 1SD United Kingdom; Oak Ridge National Laboratory, USA; North Carolina State University, USA

**Keywords:** PDBx/mmCIF, dictionary validation, structural biology, crystallography, data quality, *Visual Studio Code*, Python

## Abstract

The PDBx/macromolecular crystallographic information file (PDBx/mmCIF) framework is the standard for representing macromolecular structure data in the Protein Data Bank (PDB) and has been mandatory for crystallographic depositions since 2019. The PDBx/mmCIF dictionary, maintained by the PDBx/mmCIF working group established by the Worldwide PDB (wwPDB) consortium, defines the schema, data types, enumerations and relationships that govern the structure and content of mmCIFs. Pre-deposition validation of mmCIFs enables researchers to identify and correct errors, ensuring both dictionary compliance and that data values are meaningful and appropriate for their dataset. This validation step can significantly streamline the deposition process in the wwPDB *OneDep* system, when depositing to the PDB and Electron Microscopy Data Bank archives or using the PDB-IHM deposition system for integrative/hybrid structures, reducing delays and improving data quality. We present the *mmCIF Validator*, a comprehensive validation tool available in two complementary implementations: a *Visual Studio Code* extension for real-time interactive validation during file editing; and a standalone Python script for command-line use, batch processing, and integration into automated workflows and CI/CD (continuous integration and continuous delivery/deployment) pipelines. The validator performs comprehensive checks including item-definition validation, mandatory-item presence (with category-aware checking), enumeration-value validation, data-type validation (including automatic regex-based validation extracted from *OneDep* deposition-specific categories in the dictionary for types like email, phone, ORCID ID and PDB ID), range constraints with distinction between strictly allowed and advisory boundary conditions, parent/child category relationships, foreign-key integrity, composite-key validation for relationships defined by multiple items, duplicate category and item detection, and complex operation-expression parsing. The tool requires Python 3.7+ and uses only the Python standard library (no *pip* packages required). It works out of the box with automatic dictionary downloading from the official wwPDB repository, or users can employ a custom distant or local dictionary file. The two implementations share the same validation engine, ensuring consistent results across different usage scenarios. The validator is particularly valuable for structural biologists preparing structures for deposition, biocurators ensuring data quality and software developers building automated quality-control workflows.

## Abstract 2

The *mmCIF Validator* provides real-time automated validation of mmCIFs against a valid PDBx/mmCIF dictionary. The validator is available as both a *Visual Studio Code* extension and a standalone Python script for integration into automated workflows.

## 1. Introduction

The PDBx/macromolecular crystallographic information file (PDBx/mmCIF) framework (Westbrook *et al.*, 2022 ▸) is the standard for representing macromolecular structure data in the Protein Data Bank (PDB) (wwPDB consortium, 2019 ▸) and related databases. Since 2019, the PDBx/mmCIF format has been mandatory for depositing experimentally determined macromolecular structures by X-ray crystallography (Adams *et al.*, 2019 ▸). The PDBx/mmCIF framework was developed to address the increasing complexity and diversity of structural biology data and provides a flexible extensible system capable of accommodating large structures, complex chemistries, and data from emerging experimental methods such as cryo-electron microscopy and integrative/hybrid modelling (Westbrook *et al.*, 2022 ▸). As the structural biology community continues to generate increasingly complex data, the need for easily accessible comprehensive validation tools that ensure data quality, consistency and compliance with the dictionary schema has become critical.

Validation is a fundamental component of the PDBx/mmCIF ecosystem, enabling depositors to verify their data before submission (Westbrook *et al.*, 2022 ▸). The *OneDep* system (Young *et al.*, 2017 ▸) is the unified Worldwide PDB (wwPDB) system for deposition, biocuration and validation of macromolecular structures for the PDB and Electron Microscopy Data Bank (EMDB) archives, providing a single interface for depositors across all wwPDB partner sites [RCSB PDB, Protein Data Bank in Europe (PDBe), Protein Data Bank Japan (PDBj) and EMDB]. While files are annotated and standardized during the deposition and annotation process in *OneDep*, pre-deposition validation helps streamline the workflow by identifying dictionary-compliance issues early, before deposition. This validator focuses on dictionary-driven semantic checks and does not replace downstream scientific or structural validation performed during deposition. Various validation tools are available in different programming languages (see https://mmcif.wwpdb.org/docs/software-resources.html).

While validation tools exist, there is a gap in tools that integrate seamlessly into modern development workflows with features such as real-time validation during file editing, immediate feedback with clear error messages, integration into existing development environments, and support for automated validation in batch processing and CI/CD (continuous integration and continuous delivery/deployment) pipelines with machine-readable output formats. This need is particularly acute for institutions processing large numbers of structures and for individual researchers preparing complex structure models, where validation errors may only be discovered late in the deposition process, leading to delays and increased workload. We present the *mmCIF Validator*, a comprehensive validation tool that addresses these needs through two complementary implementations: a *Visual Studio Code* (*VS Code*) extension for interactive editing with real-time feedback, and a standalone Python script for automation and batch processing. The tool is primarily intended for power users (pipeline developers, biocurators and software developers), while remaining optional for individual depositors, who in many cases submit mmCIFs generated directly by the refinement software. A particularly valuable feature for depositors is that the validator automatically extracts and applies regex-based validation patterns from deposition-specific categories in the dictionary (such as email, phone, ORCID ID and PDB ID), ensuring that values entered during deposition preparation meet the same validation requirements as are enforced by the *OneDep* system.

Previous efforts have been made to provide validation tools for CIFs. The vscode-cif extension (Helttunen & Kainulainen, 2025 ▸) provides syntax highlighting and validation of CIF syntax and file information against CIF dictionaries, with support for macromolecular dictionaries maintained by the wwPDB. While the extension developed by Helttunen & Kainulainen (2025 ▸) focuses primarily on CIF dictionary validation, the *mmCIF Validator* is specifically tailored for the PDBx/mmCIF dictionary and provides granular validation capabilities, such as distinguishing between strictly allowed and advisory boundary conditions in range validation, and handling complex validation scenarios such as operation expressions used in virus assemblies.

## 2. Features and capabilities

The validator implements comprehensive validation checks covering multiple aspects of PDBx/mmCIF compliance. Item-definition validation verifies that all items used in the mmCIF are defined in the dictionary. Mandatory-item validation checks that all mandatory items are present, with category-aware validation that only checks mandatory items for categories that exist in the file. This ‘category-aware’ approach recognizes that different experiment types (*e.g.*X-ray crystallography, NMR spectroscopy, cryo-electron microscopy) use different sets of categories, and the validator only enforces mandatory-item requirements for categories that are present in the file, rather than requiring all possible categories regardless of the experiment type.

The validator distinguishes between errors and warnings depending on the severity of the constraint violation. Table 1 ▸ summarizes the reported errors and warnings.

Enumeration-value validation ensures that item values match the allowed enumerations in the dictionary. Enumeration violations are reported as errors since values must match the controlled vocabulary. Data-type validation automatically extracts and applies regex patterns from deposition-specific categories in the dictionary (item_type_list.construct) for types such as email, phone, ORCID ID, PDB ID and fax, ensuring that values entered during deposition preparation meet the validation requirements enforced by the *OneDep* system. The validator also implements hardcoded validations for common types, including date formats (yyyy-mm-dd, yyyy-mm-dd:hh:mm, yyyy-mm-dd:hh:mm-flex), numeric types (int, positive_int, float, float-range) and boolean types.

Range validation checks that numeric values fall within specified minimum/maximum ranges, with the validator distinguishing between two types of boundary conditions: strictly allowed boundary conditions defined in item_range, where violations are reported as errors, and advisory boundary conditions defined in pdbx_item_range, where violations are reported as warnings. This distinction is important because advisory ranges are used during the deposition process in *OneDep*, allowing depositors to identify values that, while technically within allowed ranges, may require attention or explanation. This allows users to clearly distinguish between values outside allowed ranges (which must be corrected) and values within allowed ranges but outside advisory ranges (which may require review or justification during deposition).

Parent/child-category validation verifies that when a child category is present its parent category is also present; for example, if entity_src_nat is present, entity must also be present. Foreign-key integrity validation ensures that foreign-key values in child items exist in their parent items; for example, by verifying that entity_src_nat.entity_id values must exist in entity.id. Composite-key validation extends this check to relationships where multiple items together form a composite foreign key. For example, in pdbx_entity_poly_domain, the combination of begin_mon_id + begin_seq_num must match a row in entity_poly_seq where mon_id + num appear together. The validator groups relationships by link_group_id from the dictionary and validates composite keys by checking that the tuple of child-item values matches a corresponding tuple in the parent category, ensuring data integrity for complex multi-item relationships. A special case handled by the validator includes categories such as struct_conn (covalent and other inter-atomic connections), pdbx_struct_conn_angle and geom_* (geometry restraints and geometry measurements), atom_site_anisotrop (anisotropic displacement parameters), and others that have composite keys including both the label (PDBx/mmCIF-specific items) and auth (author-provided items) fields referencing the atom_site category. The validator handles these by first attempting validation using label fields (if complete) and then falling back to auth fields when label fields are incomplete (*e.g.* when label_seq_id is missing for non-polymer entities). When auth_atom_id is absent from the file, the validator uses label_atom_id for auth validation. This ensures atoms referenced in these categories are properly validated against atom_site even when some fields are missing, catching synchronization issues that would otherwise go unnoticed.

Duplicate category and item detection identifies dictionary-breaking cases where a category or item is repeated. The validator tracks both loop blocks (from loop_ directives) and frame blocks (contiguous item–value pairs of the same category), and reports each duplicate category once and each duplicate item with the line of first occurrence.

Operation-expression validation parses and validates ‘operation expressions’ that describe how asymmetric units are transformed into biological assemblies, such as (1), (1, 2, 5), (1-4), (1, 2)(3, 4) and (X0)(1-5, 11-15), ensuring all ref­erenced operation IDs exist in pdbx_struct_oper_list.id. This capability is particularly important for virus assemblies where expressions like (1-60) reference multiple operations.

The *VS Code* extension provides real-time validation as files are edited, with automatic validation on file open, save and changes (1 s debounce). Errors and warnings are highlighted directly in the editor with precise positioning that identifies the exact problematic value, even within loop structures.

Full syntax highlighting is provided for mmCIFs. Hover information displays tag names and data-block context. Both features were adapted from the vscode-cif extension (Helttunen & Kainulainen, 2025 ▸).

The extension requires no configuration and automatically downloads the dictionary from the official wwPDB URL (https://mmcif.wwpdb.org/dictionaries/mmcif_pdbx_v50.dic/Index/), caching it locally for one month to balance dictionary freshness with download efficiency. Dictionary updates are usually released in conjunction with *OneDep* software releases, with an average update frequency of approximately one and a half months (based on an analysis of eight recent dictionary releases). The script has no external dependencies and uses only the Python standard library. It supports both local dictionary files and downloading from a URL, and provides exit codes for automated workflows (0 for success, 1 for errors).

The standalone Python script also provides a command-line interface for batch processing with enhanced JSON output that includes precise character positions and column indices for programmatic error handling, making it suitable for CI/CD pipeline integration. The script can in addition be used as a Python library (*e.g.* in workflows) and is distributed as the PyPI package pdbe-mmcif-validator. The JSON output format includes line numbers, item names, error messages, severity levels (error or warning) and precise character positions for highlighting problematic values. For example,
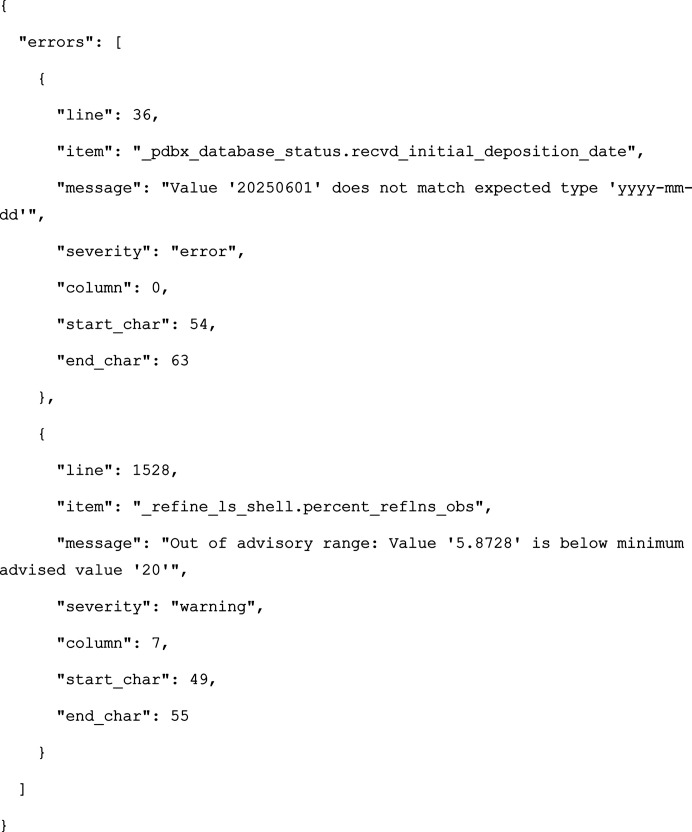


## 3. Technical implementation

Both the *VS Code* extension and the standalone Python script implementations use the same validation engine (validate_mmcif.py), ensuring consistency between interactive and automated validation. The validator consists of three main components, with the validation engine organized into separate modules for dictionary parsing, mmCIF parsing and validation logic. The *DictionaryParser* parses mmCIF dictionary files (.dic format), extracting item definitions, enumerations, range constraints, parent/child relationships, type regex patterns and category structure. The *mmCIFParser* parses mmCIFs, extracting data blocks, loop structures, item–value pairs with line numbers, category membership, and lists of loop blocks and frame blocks (each with start line, category and item names). The *mmCIF Validator* performs validation checks using the parsed dictionary and mmCIF data.

The *mmCIFParser* processes mmCIFs through a line-by-line parsing approach that handles the format’s complexity. It identifies data blocks (starting with data_), recognizes loop structures (starting with loop_), and parses both loop data and standalone item–value pairs. The parser handles multi-line strings delimited by semicolons, accumulates values that span multiple lines within loop rows, and tracks precise line numbers and column positions for each value. This enables accurate error reporting with exact character positions, even when values appear multiple times on the same line or when loop rows span multiple lines. The parser processes only the first data block in files containing multiple data blocks, which is the standard use case for mmCIF coordinate files submitted to the PDB.

The validator handles parent–child relationships defined in pdbx_item_linked_group_list, where each relationship specifies a single child item and a single parent item. The dictionary’s link_group_id field is used to group relationships that form composite keys. The validator groups relationships by (child_category, parent_category, link_group_id) and handles them differently according to the number of items in each group. For single-item relationships, each child-item-to-parent-item relationship is validated independently. For multi-item relationships (composite keys), the validator builds an index of parent rows as tuples of parent-item values and validates that the tuple of child-item values matches a corresponding tuple in the parent category. This ensures that combinations such as begin_mon_id + begin_seq_num in pdbx_entity_poly_domain cor­rectly match mon_id + num pairs in entity_poly_seq, rather than validating each item independently, which could miss cases where individual values exist but not as the required combination. During live editing in the *VS Code* extension, parent–child relationship validation is performed, re-validating the entire file. When a user edits a file, the extension triggers validation after a 1 s debounce period, re-running the complete validation process on the entire file. The validation builds maps of category membership and item values and then iterates through all parent–child relationships defined in the dictionary to perform these checks. While this approach re-validates the entire file on each edit, it ensures consistency and correctness of relationships across the file, and the 1 s debounce prevents excessive validation during rapid typing.

A key technical feature is the parsing of complex operation expressions used in assembly definitions. Unlike standard foreign-key relationships that can be validated through dictionary-defined parent/child relationships, the relationship between pdbx_struct_assembly_gen.oper_expression and pdbx_struct_oper_list.id cannot be validated using a simple foreign-key constraint due to the complex nature of operation expressions, particularly in virus assemblies, where expressions such as (1-60) reference 60 operations. The validator implements a specialized parser that handles simple expressions such as 1 and (1), comma-separated lists like (1, 2, 5), ranges such as (1-60), multiple groups like (1, 2)(3, 4), and complex expressions with transformations such as (X0)(1-5, 11-15). The parser extracts all referenced operation IDs from these complex expressions and validates that each exists in pdbx_struct_oper_list.id, providing essential validation for virus assemblies and other complex structures, where standard foreign-key validation is insufficient.

The validator parses the DDL2-based dictionary format (Bourne *et al.*, 2005 ▸), extracting information from save blocks for items and categories. It handles enumerations with or without detail fields, regex patterns embedded in type definitions and parent/child relationships defined in pdbx_item_linked_group_list.

## 4. Usage examples

The extension can be installed from the *Visual Studio Code* Marketplace or the Open VSX Registry (https://open-vsx.org), or by downloading a pre-built .vsix from the project’s GitHub Releases page. Once installed, opening any .cif triggers automatic validation with errors highlighted in the editor. Users can hover over values to see tag names and context.

For command-line use, the standalone Python script can be invoked as follows:
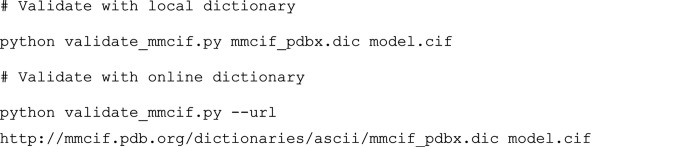


Example output:
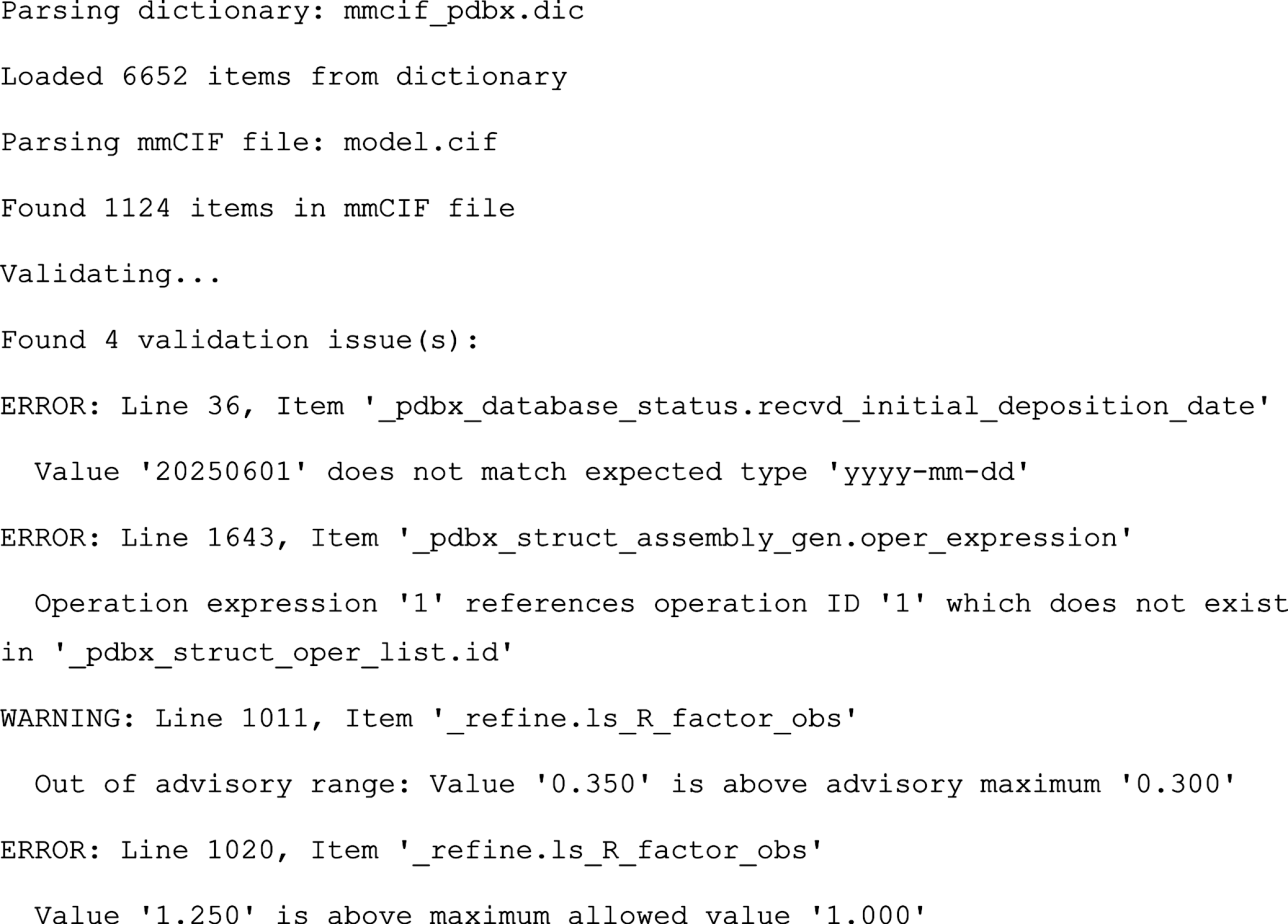


## 5. Performance and limitations

The validator is designed for practical use with mmCIFs at any stage of the structure-solving pipeline. Any mmCIF can be validated; by default, the validator uses the latest released version of the wwPDB PDBx/mmCIF dictionary to provide the validation rules and detailed feedback. This includes both coordinate-model mmCIFs and structure-factor CIFs when validated against the wwPDB dictionary. Users can specify a custom dictionary URL (*e.g.* via the script’s --url option) or use a local dictionary file (*e.g.* for offline environments, internal testing or validation against a draft/extended dictionary before official release). The dictionary parsing typically completes in a few seconds for standard dictionary files (∼170 000 lines). In the current implementation, dictionary parsing is performed for each validation run (*i.e.* per script invocation), rather than being retained as a parsed in-memory object across runs. The validation of typical mmCIFs (1000–2000 items) completes in under a second. On a representative set of mmCIFs, the built-in parser runs at ∼2.5 s per MB for full validation (parsing and checking). Validation is currently performed sequentially, and no multithreading is implemented in batch-processing mode. For large files, the *VS Code* extension allows users to increase the validation timeout in the extension settings (default 60 s, configurable up to 10 min) to avoid premature timeout messages.

The current limitation of the validator is that it processes only the first data block in files containing multiple data blocks. This is typically sufficient for standard coordinate files submitted to the PDB, where the primary structural model is in the first block, but it can be limiting for specialized multi-block files such as bundled/derived datasets that store additional blocks.

## 6. Conclusions

The *mmCIF Validator* provides a comprehensive solution for validating mmCIFs against the official dictionary, offering both interactive and automated validation capabilities. The dual implementation approach (*VS Code* extension and standalone script) makes it suitable for individual researchers preparing structures for deposition and for institutions implementing automated quality-control workflows.

## Figures and Tables

**Table 1:** Validation errors and warnings reported by the *mmCIF Validator*

Severity	Validation issue	Description
Error	Missing mandatory items	Required categories or items that are missing from the file
Error	Enumeration violations	Values that do not match the controlled vocabulary/enumeration list
Error	Data-type mismatches	Values that do not match their expected data type (*e.g.* invalid date format, non-numeric value for integer type)
Error	Strictly allowed range violations	Values outside the strictly allowed boundary conditions (item_range)
Error	Parent category missing	Child categories are present, but their required parent categories are missing
Error	Foreign-key integrity violations	Foreign-key values that do not exist or do not match in their parent items
Error	Composite-key violations	Combinations of multiple child items that are not present or do not match corresponding combinations in parent categories (including label/auth field combinations)
Error	Invalid operation-expression references in the assembly categories	Operation expressions in pdbx_struct_assembly_gen referencing operation IDs that do not exist in pdbx_struct_oper_list
Error	Duplicate category	Same category appearing more than once
Error	Duplicate item	Same item name appearing more than once
Warning	Undefined items	Items used in the mmCIF that are not defined in the dictionary (only for items not starting with _)
Warning	Advisory range violations	Values outside the advisory boundary conditions (pdbx_item_range) but within the allowed range

## Data Availability

The *mmCIF Validator* is freely available under the MIT License. The source code, documentation and issue tracker are hosted on GitHub at https://github.com/PDBeurope/mmcif-validator. The *VS Code* extension can be installed from the *Visual Studio Code* Marketplace (https://marketplace.visualstudio.com/items?itemName=PDBeurope.pdbe-mmcif-validator), from the Open VSX Registry (https://open-vsx.org/extension/PDBEurope/pdbe-mmcif-validator) or by downloading a pre-built .vsix from GitHub Releases (https://github.com/PDBeurope/mmcif-validator/releases). The standalone Python script is available in the repository; it can also be installed as the package pdbe-mmcif-validator from PyPI (https://pypi.org/project/pdbe-mmcif-validator/) and used as a library in addition to the command line.
